# Detection of Recurrent Prostate Cancer With ^18^F-Fluciclovine PET/MRI

**DOI:** 10.3389/fonc.2020.582092

**Published:** 2020-12-23

**Authors:** Kirsten Margrete Selnæs, Brage Krüger-Stokke, Mattijs Elschot, Håkon Johansen, Per Arvid Steen, Sverre Langørgen, Bjørg Yksnøy Aksnessæther, Gunnar Indrebø, Torill Anita Eidhammer Sjøbakk, May-Britt Tessem, Siver Andreas Moestue, Heidi Knobel, Torgrim Tandstad, Helena Bertilsson, Arne Solberg, Tone Frost Bathen

**Affiliations:** ^1^Department of Circulation and Medical Imaging, Faculty of Medicine and Health Sciences, NTNU – Norwegian University of Science and Technology, Trondheim, Norway; ^2^Department of Radiology and Nuclear Medicine, St. Olavs Hospital, Trondheim University Hospital, Trondheim, Norway; ^3^Department of Oncology, Ålesund Hospital, More and Romsdal Hospital Trust, Ålesund, Norway; ^4^The Cancer Clinic, St. Olavs Hospital, Trondheim University Hospital, Trondheim, Norway; ^5^Department of Cancer Research and Molecular Medicine, Faculty of Medicine and Health Sciences, NTNU – Norwegian University of Science and Technology, Trondheim, Norway; ^6^Department of Urology, St. Olavs Hospital, Trondheim University Hospital, Trondheim, Norway

**Keywords:** fluciclovine F-18, magnetic resonance imaging, positron-emission tomography (PET), prostatic neoplasms, PSA persistence and BCR, MRI, 18F-PET, PET/MRI

## Abstract

**Objective:**

Simultaneous PET/MRI combines soft-tissue contrast of MRI with high molecular sensitivity of PET in one session. The aim of this prospective study was to evaluate detection rates of recurrent prostate cancer by ^18^F-fluciclovine PET/MRI.

**Methods:**

Patients with biochemical recurrence (BCR) or persistently detectable prostate specific antigen (PSA), were examined with simultaneous ^18^F-fluciclovine PET/MRI. Multiparametric MRI (mpMRI) and PET/MRI were scored on a 3-point scale (1-negative, 2-equivocal, 3-recurrence/metastasis) and detection rates (number of patients with suspicious findings divided by total number of patients) were reported. Detection rates were further stratified based on PSA level, PSA doubling time (PSAdt), primary treatment and inclusion criteria (PSA persistence, European Association of Urology (EAU) Low-Risk BCR and EAU High-Risk BCR). A detailed investigation of lesions with discrepancy between mpMRI and PET/MRI scores was performed to evaluate the incremental value of PET/MRI to mpMRI. The impact of the added PET acquisition on further follow-up and treatment was evaluated retrospectively.

**Results:**

Among patients eligible for analysis (n=84), 54 lesions were detected in 38 patients by either mpMRI or PET/MRI. Detection rates were 41.7% for mpMRI and 39.3% for PET/MRI (score 2 and 3 considered positive). There were no significant differences in detection rates for mpMRI versus PET/MRI. Disease detection rates were higher in patients with PSA≥1ng/mL than in patients with lower PSA levels but did not differ between patients with PSAdt above versus below 6 months. Detection rates in patients with primary radiation therapy were higher than in patients with primary surgery. Patients categorized as EAU Low-Risk BCR had a detection rate of 0% both for mpMRI and PET/MRI. For 15 lesions (27.8% of all lesions) there was a discrepancy between mpMRI score and PET/MRI score. Of these, 10 lesions scored as 2-equivocal by mpMRI were changed to a more definite score (n=4 score 1 and n=6 score 3) based on the added PET acquisition. Furthermore, for 4 of 10 patients with discrepancy between mpMRI and PET/MRI scores, the added PET acquisition had affected the treatment choice.

**Conclusion:**

Combined ^18^F-fluciclovine PET/MRI can detect lesions suspicious for recurrent prostate cancer in patients with a range of PSA levels. Combined PET/MRI may be useful to select patients for appropriate treatment, but is of limited use at low PSA values or in patients classified as EAU Low-Risk BCR, and the clinical value of ^18^F-fluciclovine PET/MRI in this study was too low to justify routine clinical use.

## Introduction

Radical prostatectomy (RP) and radical external beam radiation therapy (EBRT) constitute the cornerstones of curative prostate cancer treatment. However, following these interventions, approximately one third of patients experience either biochemical recurrence (rising levels of prostate specific antigen, PSA, with time) or persistently elevated PSA levels after treatment, indicating lack of cure (1, 2). Patients with loco-regional recurrence may be candidates for curative intended salvage therapy. Retrospective studies and meta-analyses suggest improved biochemical disease control rates when local salvage treatment is initiated at low PSA levels (3, 4). Accordingly, European Association of Urology (EAU) guidelines recommend starting post-RP salvage EBRT at PSA values <0.4 ng/mL (5). On the other hand, patients with distant metastasis are not considered candidates for salvage therapy with a curative intent, although metastasis-directed therapy in patients with oligometastasis has received increased attention recently (6). Local salvage treatment (salvage EBRT after primary RP or salvage RP/lymph node dissection after EBRT) is potentially associated with rectal and genitourinary side effects. Thus, selection of eligible patients with only loco-regional disease for such interventions is crucial to avoid unnecessary overtreatment.

Conventional imaging modalities such as magnetic resonance imaging (MRI) and bone scintigraphy lack sensitivity in detecting nodal and skeletal metastases at low PSA levels (7). For post-RP staging in patients with biochemical recurrence, current EAU guidelines (5) therefore recommend prostate-specific antigen membrane (PSMA) positron emission tomography (PET)/computed tomography (CT) when PSA is >0.2 ng/mL, but only if the result potentially will influence treatment decisions (weak level of evidence). For patients with persistent PSA after initial therapy, the PSA cut-off to perform PSMA PET/CT to rule out metastatic disease is >0.2 ng/ml (weak level of evidence) (5). Alternatively, if PSMA PET/CT is unavailable and PSA is ≥1 ng/mL, choline PET/CT or ^18^F-fluciclovine PET/CT could be performed (5). After EBRT, multiparametric MRI (mpMRI) and PET/CT with a PSMA ligand, choline or ^18^F-fluciclovine are recommended in patients eligible for salvage treatment (strong level of evidence) (5). ^18^F-fluciclovine (anti-1-amino-3-18F-fluorocyclobutane-1-carboxylic acid, ^18^F-fluciclovine, also known as ^18^F-FACBC) is an amino acid tracer that is approved by the United States Food and Drug Administration (FDA) and the European Medicines Agency to detect recurrent prostate cancer, however its diagnostic performance when used in simultaneous PET/MRI is not yet determined (8, 9).

Simultaneous PET/MRI has the potential to improve the detection accuracy in recurrent prostate cancer, compared to other imaging modalities, since it combines the excellent soft-tissue contrast of MRI with the high molecular sensitivity of PET in one imaging session. Furthermore, improved detection accuracy could improve patient selection for salvage therapy and guide personalized treatment. We therefore conducted a prospective trial to evaluate detection of recurrent prostate cancer by simultaneous ^18^F-fluciclovine PET/MRI with a special focus on low PSA levels (≤1ng/mL). The detection rates were compared between combined PET/MRI and mpMRI-only, which was the current clinical standard at the time this trial was conducted.

## Materials and Methods

### Patient Inclusion

All patients had received treatment with curative intent, either RP or EBRT with 78Gy in 2 Gy fractions. Following RP, patients either had persistently detectable PSA values post-surgery, defined as at least two consecutive PSA measurements ≥ 0.2 ng/mL, or biochemical recurrence defined as two consecutive PSA measurements ≥0.2 ng/mL rising from a previous nadir of undetectability. Following EBRT, recurrence was defined as a rising PSA ≥2.0 ng/mL above the post-EBRT nadir. All patients were potential candidates for salvage treatment. Exclusion criteria were general contraindications to MRI, or hormonal treatment during the last three months. Patients with biochemical recurrence (BCR) were retrospectively categorized as EAU Low-Risk BCR (PSAdt >1 year AND pathological International Society of Urologic Pathologists (ISUP) grade < 4 for RP, interval to biochemical failure > 18 months AND biopsy ISUP grade < 4 for EBRT) or EAU High-Risk BCR (PSAdt ≤ 1 year OR pathological ISUP grade 4-5 for RP, interval to biochemical failure ≤ 18 months OR biopsy ISUP grade 4-5 for EBRT) (5, 10). The study was approved by the Regional Committee for Medical and Health Research Ethics, REC North, Norway (identifier 2015/163) and registered in clinicaltrials.gov (NCT02562131). Written and oral informed consent were collected from all patients.

### Positron Emission Tomography/Magnetic Resonance Imaging Protocol

**Figure 1:**
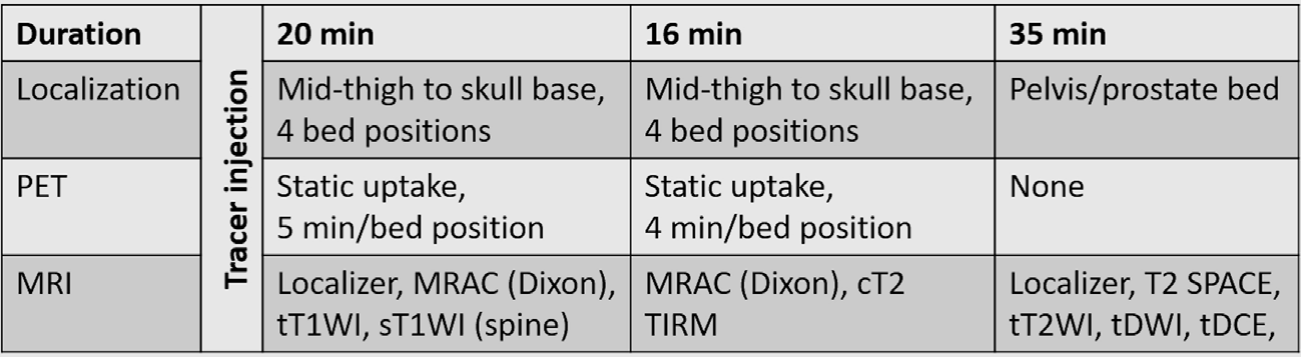
Schematic overview of the PET/MRI protocol. t, transverse plane; c, coronal plane; s, sagittal plane; T2WI, T2-weighted imaging; DWI, diffusion-weighted imaging; MRAC, MR for attenuation correction; T1W, T1-weighted imaging; T2 TIRM, T2-weighted turbo inversion recovery magnitud; T2 SPACE, T2-weighted 3D turbo spin echo; DCE-MRI, dynamic contrast enhanced MRI.

Patients were examined with simultaneous ^18^F-fluciclovine PET/MRI (3T Biograph mMR, Siemens Healthineers) at St. Olavs Hospital, Trondheim University Hospital, from September 2015 to September 2017. An overview of the imaging protocol is given in **Figure 1**, and detailed acquisition parameters are listed in **Supplementary Table S1**. ^18^F-fluciclovine, 370 MBq followed by saline flush, was manually injected while the patient was lying on the scanner table. Image acquisition started on average 3–4 min after tracer injection, to allow time for saline flush, the radiographer leaving the scanner room and MR shimming routine. Two rounds of whole-body simultaneous PET and MRI were performed, covering mid-thigh to skull base in four bed positions (caudal to cranial direction). In the first round, 5 min per bed position of PET acquisition was combined with MR for attenuation correction (MRAC) and transversal and sagittal T1-weighed imaging. In the second round, 4 min per bed position of PET acquisition was combined with MRAC and coronal T2-weighted turbo inversion recovery magnitude (T2 TIRM) imaging. Finally, MRI only (T2-weighted imaging, diffusion-weighted imaging, and dynamic contrast enhanced imaging) was performed covering the pelvis and prostate bed.

### Image Interpretation

mpMRI images were reviewed by a board-certified radiologist (S.L.) or a radiology resident (B. K.-S.), with respectively 6 and 1.5 years of experience reading prostate mpMRI, who were blinded to PET data. Images were scored on a 3-point scale at the radiologists’ discretion: 1-negative, 2-equivocal, or 3-recurrence/metastasis. Difficult cases were discussed between the readers and consensus reached. Subsequently, a nuclear medicine physician (H. J.), with 3 years of experience reading PET images, reviewed PET images with MRI as anatomical background and with access to the radiologist’s mpMRI evaluation. PET findings were scored as 1-negative, 2-equivocal, or 3-recurrence/metastasis based on elevated tracer uptake compared to background. Lesions scored as 2-equivocal had slightly increased tracer uptake while lesions scored as 3-recurrence/metastasis had clearly elevated tracer uptake compared to background. PET findings were to be scored independently of mpMRI findings, i.e., a lesion with mpMRI score 3-recurrence/metastasis was to get a PET score 1-negative if the ^18^F-fluciclovine uptake was not elevated compared to background. In a final step, the radiology resident (B. K.-S.) reviewed all examinations a second time, with access to the initial individual scores. A combined PET/MRI score was then generated based on the full mpMRI examination and co-registered PET images.

### Patient Follow-Up

After the PET/MRI examination the results for each patient were discussed in multi-disciplinary team meetings where urologists, oncologists, radiologists and nuclear medicine physicians would give a recommendation for further follow-up or treatment. There was no routine follow-up of patients (PSA measurements, histology or imaging) as part of the project.

## Evaluation of Added Value of the Positron Emission Tomography Acquisition

To better understand the incremental value of PET/MRI to mpMRI and to explore the impact on patient management of the added PET examination, we performed a subgroup analysis of the cases where the PET/MRI score differed from the score based on mpMRI only. An experienced oncologist (A.S.) retrospectively reviewed the case files and the comments from the multi-disciplinary team meetings for this subgroup to investigate whether the PET acquisition had affected the follow-up and treatment compared to the decision that would have been made based on mpMRI alone.

### Statistical Analysis

Detection rates for mpMRI and combined PET/MRI were calculated as the number of patients with suspicious findings divided by the total number of patients scanned. PSA doubling time (PSAdt) was calculated based on patients’ available PSA measurements prior to the PET/MRI examination, using the online PSAdt calculator of the Radiation Therapy Oncology Group Foundation (https://www.rtog.org/ClinicalTrials/CalculationofPSADoublingTimePSADT.aspx, accessed April 2018). Detection rates were stratified based on PSA level, PSAdt at the time of imaging, primary treatment, and patient category. The significance of differences in detection rates between mpMRI only and PET/MRI was determined with the McNemar’s test. Association in detection rates between subgroups of patients (based on PSA level, PSAdt, primary treatment and patient category) were tested with the chi-square test. Median PSA levels and PSAdt in patients with and without lesions with elevated tracer uptake (scored as 2-equivocal or 3 – recurrence/metastasis by the nuclear medicine physician) were compared with the Mann-Whitney U test. When appropriate, the false discovery rate approach described by Benjamini and Hochberg (11) was used to correct for multiple testing. p-values (or adjusted p-values) <0.05 were considered significant. Unless otherwise stated, statistical analyses were performed with SPSS (IBM SPSS Statistics 25.0).

## Results

### Patient Cohort

Altogether 87 patients were included in the study, of whom three patients examined with PET/CT and a separate MRI due to instrument error were excluded from further analysis, leaving 84 evaluable patients (**Table 1**). PSAdt could only be calculated for 57 patients (67.9%) (n = 48 with radical prostatectomy as primary treatment and n = 9 with external beam radiation therapy as primary treatment) due to lack of sufficient pre-imaging PSA values (n=21) or stably elevated PSA (n=6) for the remaining. The majority of patients were included due to biochemical recurrence (n=66, 78,6%) and categorized as EAU Low-Risk BCR (n=12, 14.3%) or EAU High-Risk BCR (n=39, 46.4%) while 15 patients (17.9%) with biochemical recurrence could not be assigned to a category due to lack of PSAdt. The remaining 18 patients (21.4%) were included due to PSA persistence.

**Table 1:** Patient characteristics.

Characteristics	All evaluable patients (n = 84)	Primary RP (n = 71)	Primary EBRT (n = 13)
Age (years) Median [range]	65.4 [53.0–84.7]	65.1 [55.3–79.9]	70.8 [53–84.7]
Time from primary treatment to PET/MRI (months) Median [range]	48 [3–163]	47 [3–163]	54 [36–126]
PSA (ng/mL) at time of PET/MRI	0.7 [0.2–12.9]	0.6 [0.2–11.0]	5.2 [2.1–12.9]
Median [range]			
PSAdt (months)*	7.6 [1.2–52]	7.7 [1.5–52]	7.2 [1.2–14.1]
PSA < 1.0 ng/mL, n (%)	48 (57.1)	48 (67.6)	0 (0)
PSA ≥ 1.0 ng/mL, n (%)	36 (42.9)	23 (32.4)	13 (100)
Gleason grade group**			
Grade group 1, n (%)	1 (1.2)	1 (1.4)	0 (0)
Grade group 2, n (%)	24 (28.6)	21 (29.6)	3 (23.1)
Grade group 3, n (%)	30 (35.7)	26 (36.6)	4 (30.8)
Grade group 4, n (%)	14 (16.7)	11 (15.5)	3 (23.1)
Grade group 5, n (%)	15 (17.9)	12 (16.9)	3 (23.1)
Salvage treatment:			
ePLND, n (%) EBRT, n (%) RP, n (%) Endocrine, n (%)	27 (32.1) 24 (28.6) 1 (1.2) 22 (26.1)	25 (35.2) 24 (33.8) 0 (0) 10 (14.1)	2 (15.4) 0 (0) 1 (7.7) 12(92.3)

EBRT, external beam radiation therapy; ePLND, extended pelvic lymph node dissection; RP, radical prostatectomy. *PSAdt from the subgroup of all patients in whom this variable could be calculated (n = 57 overall, n = 48 primary RP patients, n = 9 EBRT patients). **Gleason score is from histopathology after RP if available (n = 69), otherwise from diagnostic biopsies prior to primary treatment (n = 15).

### Detected Lesions by Site and Modality

**Figure 2:**
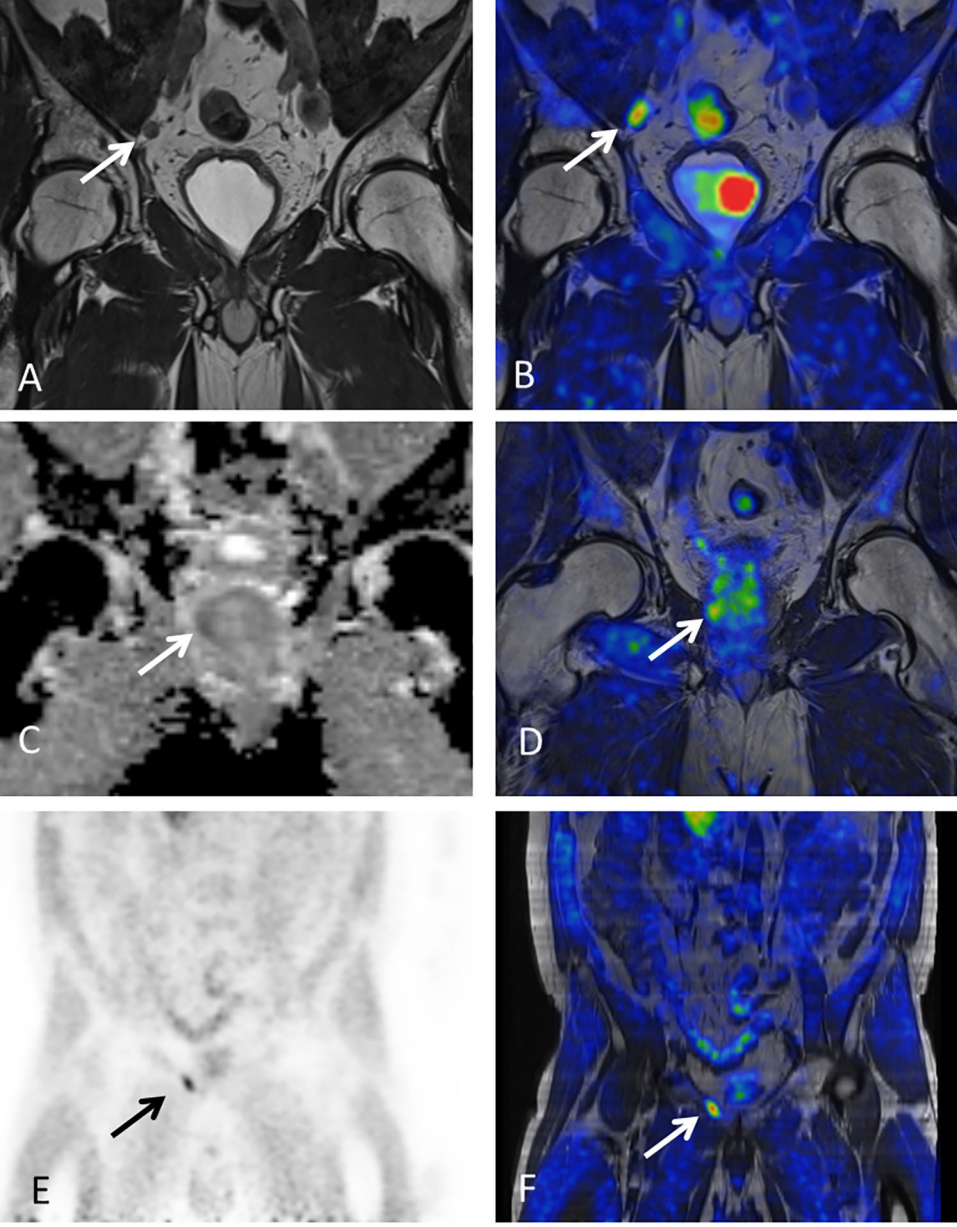
Examples of lesion visibility. Arrows point out lesions detected by ^18^F-fluciclovine PET/MRI. **(A, B)** 57-year-old patient with PSA of 0.6 ng/mL and one pelvic lymph node suspicious for metastasis based on mpMRI **(A)** and PET (maximum standardized uptake value [SUV max]=3.95) **(B). (C, D)** Prostatic lesion with restricted diffusion [Apparent diffusion coefficient (ADC)=982 x10^−6^mm^2^/s] **(C)** and increased tracer uptake (SUVmax=4.19) **(D)** in 62-year-old man with history of primary EBRT and PSA of 5.5 ng/mL at time of imaging. **(E, F)** Pelvic bone lesion (SUVmax=4.04) in 60-year-old male with PSA of 1.7 ng/ml. This lesion was initially missed by mpMRI but could be detected retrospectively.

In total, 54 lesions in 38 patients (45.2%) (median 1, range 1-4 lesions per patient) were scored as equivocal or positive (score 2 or 3) for prostate cancer recurrence by either mpMRI, PET or PET/MRI. An overview of detected lesions by site and modality is given in **Table 2** and examples of lesion visibility are shown in **Figure 2**.

**Table 2:** Detected lesions by site and modality.

Lesion site	Overall* N = 38 (24)	mpMRI N = 35 (19)	PET N = 25(16)	PET/MRI N = 33(24)
Prostate/-bed gland bed vesicle vesicle remnants	17(9) 6(3) 5(5) 1(0) 5(1)	16(8) 5(3) 5(5) 1(0) 5(0)	9(4) 5(2) 3(1) 0(0) 1(1)	14(9) 5(3) 5(5) 0(0) 4(1)
Intrapelvic nodes	27(21)	26(16)	17(15)	26(21)
Extrapelvic nodes	8(4)	4(2)	7(3)	7(4)
Bone	2(1)	1(0)	1(1)	1(1)
Total lesions	54(35)	47 (26)	34(23)	48(35)

Number of lesions scored as equivocal or positive (score 2 or 3) is presented, with number lesions classified as positive (score 3) in brackets. *Lesions scored as positive/equivocal by either mpMRI, PET, or PET/MRI.

The total number of lesions detected was similar for mpMRI only and PET/MRI combined (n = 47 and n = 48), while fewer lesions were detected by PET only (n=34). When only considering score 3 lesions, fewer lesions were detected by mpMRI and PET individually (n=26 and 23, respectively) than by combined PET/MRI (n=35). Finally, 46 patients (54.8%) had no suspicious findings, i.e., negative score on mpMRI, PET and PET/MRI.

The majority of lesions were found in the pelvic lymph nodes. Five paraaortic lymph node lesions were detected (in 4 patients) while the remaining extrapelvic lymph nodes were inguinal lymph nodes that were equivocal (score 2) due to a slightly increased tracer uptake. In the prostate/-bed region, lesions were found in the prostatic bed and seminal vesicle remnants in patients with previous RP and in the prostate and seminal vesicles in patients with primary EBRT. Only two bone lesions were detected, both in the pelvic area. No lesions were detected above the diaphragm.

### Detection Rates

**Figure 3:**
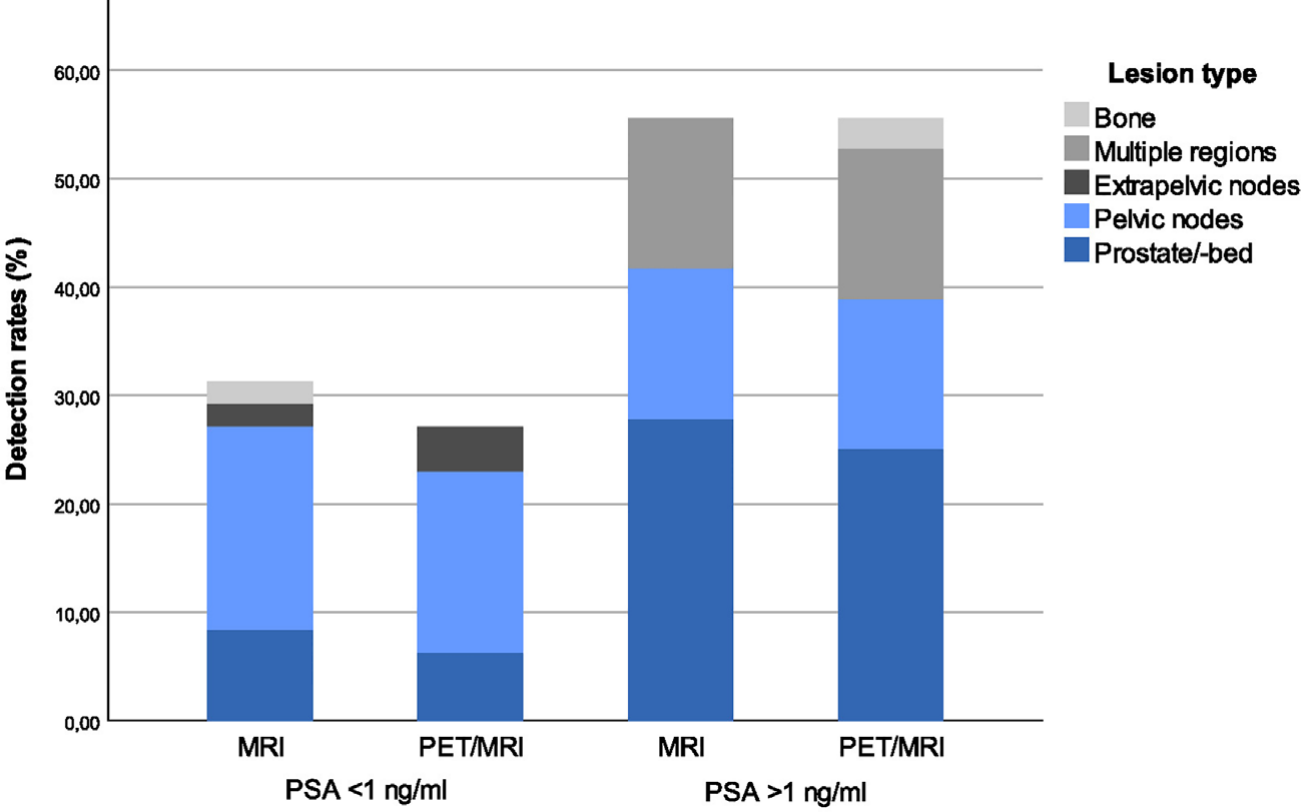
Patient-based detection rates stratified by PSA-level and lesion site. When lesion site is “multiple regions” this indicates patients with lesions in multiple regions (according to the main regions used in **Table 2**).

The detection rates were 41.7% for mpMRI and 39.3% for PET/MRI when score 2 and 3 were considered positive and 22.6% for mpMRI and 28.6% for PET/MRI when considering only score 3 as positive (**Table 3**). There were no statistically significant differences in detection rates for mpMRI only versus combined PET/MRI (p = 0.69 for score 2+3, p = 0.06 for score 3 lesions only). Patient-based detection rates (score 2 and 3 considered positive) stratified by PSA level and lesion site for mpMRI and PET/MRI are illustrated in **Figure 3**.

**Table 3:** Detection rates by PSA levels, imaging modality, primary treatment and patient categories.

Detection rates (%)	mpMRI		PET/MRI	
	Score 2+3	Score 3	Score 2+3	Score 3
Overall	41.7%	22.6%	39.3%	28.6%
**PSA level**				
PSA≥1 ng/mL PSA<1ng/mL p-value, PSA subgroup comparison Adjusted p-value	55.6% 31.3% (0.025) 0.025	36.1% 12.5% (0.01) 0.013	55.6% 27.1% (0.008) 0.013	44.4% 16.7% (0.005) 0.013
PSAdt*				
PSAdt<6months PSAdt≥6 months p-value, PSAdt subgroup comparison	41.2% 35% (0.658)	23.5% 20% (0.765)	47.1% 32.5% (0.297)	35.3% 20% (0.220)
**Primary treatment**				
RP EBRT p-value, primary treatment subgroup comparison Adjusted p-value	35.2% 76.9% (0,005) 0.02	18.3% 46.2% (0.027) 0.028	33.8% 69.2% (0.016) 0.028	23.9% 53.9% (0.028) 0.028
**Patient category**				
PSA persistence EAU Low-Risk BCR EAU High-Risk BCR BCR unassigned p-value, patient category comparison Adjusted p-value	38.9% 33.3% 48.7% 33.3% (0.653) 0.653	27.8% 0% 30.8% 13.3% (0.035) 0.070	38.9% 25.0% 48.7% 26.7% (0.323) 0.431	38.9% 0% 38.5% 13.3% (0.005) 0.020

p-values and adjusted p-values are from chi-square tests for evaluation of differences in detection rates between subgroups. *In patients for whom PSAdt could be calculated (n = 57). The group BCR unassigned are patients with biochemical recurrence that could not be assigned to one of the EAU risk groups due to lack of PSAdt.

As seen in **Table 3**, disease detection rates were significantly higher in patients with PSA ≥ 1ng/mL than in patients with lower PSA levels at time of imaging. Detection rates did not differ significantly between patients with PSAdt above versus below 6 months (**Table 3**). Detection rates (and PSA level at time of imaging) in patients with primary EBRT were higher than in patients with RP as primary treatment (**Table 3** and **Table 1**). It should be noted that EBRT patients had more advanced disease prior to initial treatment and that due to the definition of biochemical recurrence used for EBRT patients, none of those patients had PSA below 2 ng/ml at the time of imaging.

Detection rates for the patient categories *PSA persistence*, *EAU Low-Risk BCR*, *EAU High-Risk BCR* and *BCR unassigned* are listed in **Table 3**. Among patients categorized as *EAU Low-Risk BCR* there were no lesions scored as 3-recurrence/metastasis either for mpMRI or for PET/MRI (detection rate 0%).

### Prostate-Specific Antigen Metrics Related to Tracer Uptake

**Figure 4:**
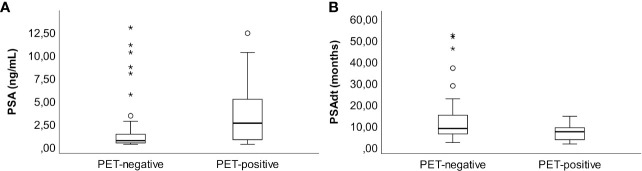
PSA (ng/mL) at time of imaging **(A)** and PSAdt (months) **(B)** in patients without versus with PET-positive lesions (score 2 or 3).

Patients with elevated ^18^F-fluciclovine uptake lesions (score 2 and 3 assigned by nuclear medicine physician) had higher median PSA levels (2.5 ng/mL, range 0.2–12.3 ng/mL), than did PET-negative patients (median PSA 0.6ng/mL, range 0.2 - 12.9 ng/mL, p=0.001) (**Figure 4A**). Furthermore, the PET-positive patients had shorter PSAdt than did PET negative patients; median PSAdt 6.9 months, range 1.2–14.1 versus 8.4, range 1.8 - 52.0 months (p=0.049, **Figure 4B**).

### Discrepancy Between Multiparametric Magnetic Resonance Imaging and Positron Emission Tomography/Magnetic Resonance Imaging Score

In total, 15 lesions (27.8 % of all lesions) from 11 patients (28.9% of patients with suspicious lesions) had a discrepancy between mpMRI and PET/MRI scores. For ten patients, this observation also meant a change in the overall patient score (n=4 down-staged from equivocal to negative, n=4 up-staged from equivocal to positive and n=2 up-staged from negative to equivocal and positive respectively). **Table 4** gives a detailed overview of lesions/patients with a score discrepancy between mpMRI and PET/MRI. Overall, ten lesions scored as equivocal (score 2) were changed to either negative (score 1, n=4) or positive (score 3, n=6), based on the added PET examination.

**Table 4:** Discrepancy between mpMRI and combined PET/MRI score.

Primary treatment	mpMRI score	PET score	PET/MRI score	Site of lesion	Consequences for choice of treatment
RP	2	1	1	Seminal vesicle	No
**RP**	2	3	3	Seminal vesicle	No
EBRT	2	1	1	Seminal vesicle	No
**RP+ePLND**	2	3	3	LN, pelvic	No
RP	2	1	1	LN - pelvic	Yes, EBRT field reduced
**RP+ePLND**	2 2	3 2	3 3	LN – pelvic LN - pelvic	Yes, EBRT field extended
**RP+ePLND, sEBRT**	1 1	2 2	2 2	LN, other LN, other	No
**EBRT**	2 1 1	3 3 3	3 3 3	LN, pelvic LN, pelvic LN, paraaortic	Yes, systemic treatment due to paraaortic LN
EBRT+ePLND	2	3	3	LN – paraaortic	No*
**RP+ePLND**	1	3	3	Bone - pelvic	Yes, extended EBRT field
RP+ePLND	2	1	1	Bone - pelvic	No

Patients in bold were up-staged based on PET. *patient had multiple other lymph nodes with score 3 on both mpMRI and PET/MRI and overall score for the patient was not changed. RP, radical prostatectomy; EBRT, external beam radiation therapy; ePLND, extended pelvic lymph node dissection; sEBRT, salvage EBRT; LN, lymph node.

### Consequences for Treatment

For four out of ten patients with discrepancy between mpMRI and PET/MRI score, the change in score affected the treatment choice (**Table 4**). In one patient, only systemic treatment was recommended rather than local salvage treatment, due to detection of a para-aortic LN with tracer uptake. For three patients, salvage EBRT fields were changed due to discordant imaging findings.

## Discussion

Our prospective study demonstrated that simultaneous ^18^F-fluciclovine PET/MRI can detect lymph node, prostatic and bone lesions suspicious for prostate cancer recurrence in patients with a wide range of PSA levels. The overall detection rate was in the ranges previously reported for ^18^F-Flucoclovine PET/CT (12) or ^11^C-Choline PET-CT (13), but was substantially lower than rates reported for PSMA-targeted radiotracers (14, 15). In 46 of 84 patients fulfilling generally accepted criteria of recurrent or residual prostate cancer (5, 16, 17) and categorized as *EAU Low-Risk BCR* (n=7), *EAU High-Risk BCR* (n=18), *BCR unassigned* (n=10) and *PSA persistence* (n=11), no suspicious lesions were found. It is still highly likely that the majority of them had undetected persistent prostate cancer and the false-negative rate of this examination is therefore probably high.

Most previous studies on ^18^F-fluciclovine PET for detection of recurrent prostate cancer have been performed using PET/CT systems. We hypothesized that simultaneous PET/MRI would improve detection accuracy of recurrence since this hybrid imaging modality combines the soft-tissue contrast of MRI with molecular sensitivity of PET. Our results showed no significant difference in overall detection rates for mpMRI only versus combined PET/MRI. However, we observed that the number of equivocal findings was reduced when MR images were evaluated in conjunction with PET uptake. When the initial score of a lesion was 3 by either modality, this finding was so convincing for the reviewer generating the combined PET/MRI score that the combined score was also 3, even if the second modality scored the lesion as negative or equivocal. Moreover, altogether 27.8% of all lesions detected had discrepant mpMRI and PET/MRI scores. The majority of these discrepancies were in lesions scored as equivocal (score2) on mpMRI and either down-staged or up-staged based on tracer uptake. There were also cases of lesions with elevated tracer uptake that had been missed by the mpMRI evaluation. These include one patient with one pelvic and one extra pelvic lymph node non-suspicious on mpMRI, but with clearly elevated tracer uptake (score 3 on PET), and one patient with elevated tracer uptake in a bone lesion (score 3 on PET) that was missed by the initial mpMRI reading, but identified as suspicious for bone metastasis retrospectively (**Figure 2C**). These observations indicate that combining ^18^F-fluciclovine PET with mpMRI may offer complementary information to that obtained with mpMRI only. Although it would have been interesting to further investigate whether the location of recurrence had an impact on which modality was the most important for the final score, there were too few lesions in our study for such analysis.

We observed lower detection rates in patients with serum PSA< 1 ng/mL at the time of imaging for mpMRI only (31.3% for PSA < 1 ng/mL versus 55.6% for PSA > 1 ng/ mL) and combined PET/MRI (27.1% for PSA < 1 ng/mL versus 55.6% for PSA > 1 ng/ mL). Evans et al (15) showed in a meta-analysis that all tracers commonly used for the imaging of prostate cancer recurrence (Choline, ^18^F-fluciclovine and PSMA) demonstrate a similar trend of decreased detection rates at lower PSA levels. Previous studies on ^18^F-fluciclovine PET/CT showed detection rates in the range of 21%-41% for PSA < 1 ng/mL (9, 18–20), whereas detection rates were up to 67% with PSMA-based tracers at these low PSA levels (15, 20, 21). It thus remains a problem that the available imaging options have relatively low detection rates at the PSA levels where salvage treatment after RP should preferably be initiated for best cancer control rates (3, 4).

Although we demonstrated that it is possible to find lesions in patients with PSA levels down to 0.2 ng/mL, it might not be cost-effective to introduce this modality in a clinical routine, unless one could carefully select subgroups of patients that are likely to benefit. In that regard, we also explored the association between detection rates and PSAdt. One might expect that patients with shorter PSAdt would have more aggressive disease and therefore a higher chance of positive imaging findings. Although we observed a shorter median PSAdt in patients with lesions (score 2 and 3) in PET images compared to patients without such PET lesions (6.9 months versus 8.4 months), we did not observe any significant differences in detection rates in patients with PSAdt over versus under 6 months. However, the number of patients in the PSAdt analysis was small and PSAdt as a variable predicting positive imaging cannot be ruled out based on this study.

The definition of biochemical recurrence following EBRT used for inclusion in this study was a rising PSA≥ 2.0 ng/mL above the post-EBRT nadir. As could be expected, the serum PSA at time of imaging was higher in EBRT patients than in RP patients. Indeed, in our patient cohort, the median PSA value among EBRT patients was 5.2 ng/mL (minimum 2.1 ng/mL) compared to 0.6 ng/mL (minimum 0.2 ng/mL) in the RP patients. By evaluating detection rates based on primary treatment, we found that the overall detection rate for PET/MRI (score 2 and 3 lesions) was significantly higher in EBRT patients than in RP patients (69.2% versus 33.8%, adjusted p=0.032). Furthermore, current EAU guidelines emphasize the need for precise localization of local recurrence after EBRT for biopsy targeting and guiding local salvage treatment, and recommend mpMRI and PET/CT (PSMA, choline or ^18^F-fluciclovine) in candidates for local salvage therapy (5). Simultaneous ^18^F-fluciclovine PET/MRI (or PET/MRI with other available tracers) could therefore be an option in patients with biochemical recurrence following EBRT. Our results should, however, be interpreted with caution since the number of EBRT patients in our cohort was rather low (n=13).

In 2019, the EAU suggested to stratify patients with biochemical recurrence into EAU Low-Risk BCR and EAU High-Risk BCR since outcome among patients with biochemical recurrence varies (5, 10), and this stratification has recently been validated in a European cohort (22). Based on this, we retrospectively categorized our patients with biochemical recurrence into these groups. Interestingly, none of the patients that were categorized as EAU Low-Risk BRC (n=12) had any lesions scored as 3 – recurrence/metastasis by mpMRI or PET/MRI. Patients with EAU Low-Risk BCR after radical prostatectomy are recommended active surveillance and possibly delayed salvage radiotherapy (5), and the low detection rate we observe in the EAU Low-Risk BCR group could also support that ^18^F-Fluciclovine PET/MRI is of limited use in this patient group. This should however be interpreted with caution due to the low number of patients assigned to the EAU Low-Risk BCR group.

More reliable localization of the site of recurrence can affect treatment decisions. To evaluate the effect on treatment decision by adding PET to the mpMRI examination, we reviewed the files of patients with discrepancy between mpMRI and PET/MRI scores. In four of ten patients in whom the final combined PET/MRI scores differed from mpMRI only scores, this discrepancy led to a change in treatment decision. EBRT fields were modified in three patients and systemic therapy was initiated in one patient due to the presence of a para-aortic lymph node lesion. Moreover, the result of the PET acquisition could also hypothetically have affected treatment choice for other patients in the sense that a negative PET score in combination with a negative mpMRI score and stable PSA values could give confidence in a decision to postpone salvage treatment.

A limitation in this study is the lack of reference standard to categorize the findings as true or false positive since very few lesions were biopsied to confirm presence and absence of disease. Another limitation is the low number of patients enrolled and that the study cohort was rather heterogeneous with a mix of primary RP and EBRT and various types of adjuvant or salvage treatment prior to imaging. Furthermore, some patients had persistent PSA after initial treatment, while others had a slowly rising PSA after several years of undetectable serum PSA. It is likely that these factors affected the detection rates and hamper direct comparison of our results to those of other studies. Finally, the setup for image interpretation could have been improved by using dual trained radiology and nuclear medicine specialists performing individual evaluations of mpMRI and PET/MRI. However, our institution did not have any dual trained specialists with competence in prostate imaging during the project period and we chose a setup that is close to the clinical practice where radiologists interpret MR images and a nuclear medicine physician interpret PET images with MR as anatomical background. At the time when this study was initiated, there were no standardized reporting guidelines for ^18^F-Fluciclovine PET published. For future studies we recommend that image interpretation follow such guidelines (23).

## Conclusion

Combined ^18^F-fluciclovine PET/MRI can detect lesions suspicious for recurrent prostate cancer following curative-intent treatment, and offer additional diagnostic information compared to mpMRI, even in patients with low PSA values. The combination of these modalities may be useful to select certain patients for appropriate treatment, but is of limited use at low PSA values or in patients classified as EAU Low-Risk BCR, and the clinical value of ^18^F-fluciclovine PET/MRI in this study population was too low to justify routine clinical use. Accordingly, new imaging methods and strategies are still needed to further improve the detection accuracy in candidates for salvage treatment.

## Data Availability Statement

The raw data supporting the conclusions of this article will be made available by the authors, without undue reservation.

## Ethics Statement

The studies involving human participants were reviewed and approved by Regional Committee for Medical and Health Research Ethics, REC North, Norway. The patients/participants provided their written informed consent to participate in this study.

## Author Contributions

KS, BK-S, ME, M-BT, SM, HB, AS, and TB conceptualized and designed the study. KS, BK-S, ME, HJ, PS, SL, BA, GI, TS, HK, TT, HB, and AS acquired the data. KS, BK-S, ME, and TB analyzed and interpreted the data. KS and BK-S drafted the manuscript. All authors critically revised and approved the final version of the manuscript. All authors contributed to the article and approved the submitted version.

## Funding

The study was supported by The Norwegian Cancer Society – project ID 100792; Movember Foundation– project ID 102050. M-BT has received funding from European Research Council (ERC) under the European Union’s Horizon 2020 research and innovation program (grant agreement No 758306).

## Conflict of Interest

The authors declare that the research was conducted in the absence of any commercial or financial relationships that could be construed as a potential conflict of interest.
