# Supplementary material for: Detection of Recurrent Prostate Cancer With 18F-Fluciclovine PET/MRI
Source: Front Oncol. 2020 Dec 23;10:582092. doi: 10.3389/fonc.2020.582092 (PMC7786298; doi:10.3389/fonc.2020.582092)
Supplement: Supplementary file 1 [file Table_1.docx]

Supplementary material

**Table S1: MRI acquisition parameters**

| **Localization** | **TA (min:sec)** | **Sequence** | **Orientation** | **TR/TE1/TE2 (ms)** | **Flip angle** | **FOV** | **Acquisition Matrix** | **No. slices** | **Resolution (mm)** | |
| --- | --- | --- | --- | --- | --- | --- | --- | --- | --- | --- |
|  |  |  |  |  |  |  |  |  | **In-plane^*^** | **Slice** |
| Whole body | 0:26 | Localizer (FastviewFoV) | Transversal | 2.56/1.44 |  | 480x420 | 96x84 |  | 5x5 | 5 |
| Thigh to skull base,  4 bed positions | 0:19 | MRAC (Dixon) | Coronal | 3.6/1.23/2.46 | 10 | 500x328 | 192x79 |  | 2.6x2.6 | 3.12 |
|  | 5:00 | PET |  |  |  |  |  |  |  |  |
|  | 2:08 | T1W TSE | Transversal | 500/9.5 | 140 | 380x380 | 384 x 230 | 50 | 1.0 x 1.0 | 4.0 |
|  | 0:58 | T1W TSE | Sagittal | 500/9.5 | 140 | 380x380 | 384 x 230 | 15 | 1.0x1.0 | 4.0 |
| Thigh to skull base,  4 bed positions | 0:19 | MRAC (Dixon) | Coronal | 3.6/1.23/2.46 | 10 | 500x328 | 192x79 |  | 2.6x2.6 | 3.12 |
|  | 4:00 | PET |  |  |  |  |  |  |  |  |
|  | 2:04 | T2 TIRM | Coronal | 2780/82 | 120 | 450x408 | 384 x 226 | 50 | 1.2 x 1.2 | 6.0 |
| Pelvis | 0:57 | Localizer |  |  |  |  |  |  |  |  |
|  | 8:35 | T2 SPACE | Coronal (3D) | 1500/82 | 110 | 320x320 | 320x320 | 144 | 1.0x1.0 | 1.0 |
|  | 1:14 | DWI (EPI) | Coronal, b=0 | 10400/67 |  | 256x251 | 100x98 | 40 | 2.6x2.6 | 4.0 |
|  | 5:24 | DWI (EPI) | Coronal, b=0, 50, 800 | 10400/67 |  | 256x251 | 100x98 | 40 | 2.6x2.6 | 4.0 |
|  | 2:31 | T2W | Sagittal^**^ | 5590/100 | 160 | 200x200 | 320x320 | 19 | 0.6x0x6 | 3.0 |
|  | 4:28 | T2W | Coronal^**^ | 5470/101 | 160 | 192x192 | 320x288 | 19 | 0.6x0.6 | 3.0 |
|  | 6:30 | T2W | Transversal | 6840/104 | 160 | 192x192 | 384x346 | 23 | 0.5x0.5 | 3.0 |
|  | 1:07 | DWI (EPI) | Transversal, b=0 | 6100/67 |  | 256x241 | 102x96 | 23 | 2.5x2.5 | 3.0 |
|  | 6:37 | DWI (EPI) | Transversal, b=0, 50, 400, 800 | 6100/67 |  | 256x241 | 102x96 | 23 | 2.5x2.5 | 3.0 |
|  | 3:37 | DCE(fl3d_vibe) | Transversal | 4.2/0.97 | 14.5 | 230x230 | 128x128 | 26 | 1.8x1.8 | 3.0 |

Note: ^*^Reconstructed in-plane resolution, ^**^only in patients with intact prostate (radiotherapy as primary treatments)
